# Osteoporosis, Fractures, and Diabetes

**DOI:** 10.1155/2014/820615

**Published:** 2014-06-23

**Authors:** Peter Jackuliak, Juraj Payer

**Affiliations:** 5th Department of Internal Medicine, Comenius University Faculty of Medicine and University Hospital in Bratislava, Ruzinovska 6, 826 06 Bratislava, Slovakia

## Abstract

It is well established that osteoporosis and diabetes are prevalent diseases with significant associated morbidity and mortality. Patients with diabetes mellitus have an increased risk of bone fractures. In type 1 diabetes, the risk is increased by ∼6 times and is due to low bone mass. Despite increased bone mineral density (BMD), in patients with type 2 diabetes the risk is increased (which is about twice the risk in the general population) due to the inferior quality of bone. Bone fragility in type 2 diabetes, which is not reflected by bone mineral density, depends on bone quality deterioration rather than bone mass reduction. Thus, surrogate markers and examination methods are needed to replace the insensitivity of BMD in assessing fracture risks of T2DM patients. One of these methods can be trabecular bone score. The aim of the paper is to present the present state of scientific knowledge about the osteoporosis risk in diabetic patient. The review also discusses the possibility of problematic using the study conclusions in real clinical practice.

## 1. Introduction

Osteoporosis is a skeletal disorder characterized by compromised bone strength predisposing to an increased risk of fractures. Many of these fractures are associated with significant morbidity and mortality. Diabetes is also an increasingly prevalent disease, with significant associated morbidity and mortality. Additionally, it has become apparent in recent years that both type 1 diabetes mellitus (T1DM) and type 2 diabetes mellitus (T2DM) are associated with an increased risk of osteoporosis-associated fractures [1–3]. Authors present the overview of factors involved in the risk of osteoporosis and fractures in both types of diabetes.

## 2. Diabetes Mellitus

Diabetes is a group of metabolic diseases characterized by hyperglycemia resulting from defects in insulin secretion, insulin action, or both. The chronic hyperglycemia of diabetes is associated with long-term damage, dysfunction, and failure of different organs. Diabetes mellitus is a common disease in most parts of the world. Worldwide 382 million people have diabetes and by 2035 this will rise to 592 million. A further 316 million with impaired glucose tolerance are at high risk from the disease—an alarming number, that is, set to reach 471 million by 2035. The number of people with type 2 diabetes is increasing in every country. Diabetes caused 5.1 million deaths in 2013; every six seconds a person dies from diabetes [4]. Well known late complications of diabetes are microvascular disease including nephropathy, retinopathy, neuropathy, and macrovascular disease such as acute coronary syndrome, claudicatio intermittens, and stroke [5]. However, the bone turnover and thus the skeletal integrity may also be affected by diabetes, and diabetic bone disease can represent an overlooked complication of diabetes [6].

## 3. Osteoporosis

Osteoporosis is defined as a combination of reduced bone mass and altered bone quality, with microarchitectural abnormalities, resulting in decreased bone strength with an increased risk of fractures [7]. Based on the present definition, both bone density and quality, which encompass the structural and material properties of bone, are important factors in the determination of bone strength. Twenty-two million women and 5,5 million men in the 27 countries of the European Union (EU27) were estimated to have osteoporosis, and 3,5 million new fragility fractures were sustained, comprising 610,000 hip fractures, 520,000 vertebral fractures, 560,000 forearm fractures, and 1,800,000 other fractures (i.e., fractures of the pelvis, rib, humerus, tibia, fibula, clavicle, scapula, sternum, and other femoral fractures) [8].

At present, the diagnosis of osteoporosis rests on areal bone mineral density (BMD) measurement using dual-energy X-ray absorptiometry (DXA). The results are reported as the difference, in standard deviations (SDs), with the peak bone mass (*T*-score). The World Health Organization (WHO) defines osteoporosis as a BMD *T*-score of −2.5 or less [9]. Low BMD has been recognized as a good predictor of osteoporotic fracture risk [10]. Nevertheless, although widely used, a major limitation of BMD measurement is that a substantial degree of BMD overlap exists between subjects with and without subsequent fractures [11, 12]. An additional explanation for this is that BMD does not capture all of the factors that contribute to bone strength. Among these factors is trabecular bone microarchitecture, which also appears to be a significant determinant of bone strength and is complementary to bone density [13, 14]. Another limitation of BMD measurements is that they disproportionately evaluate cortical bone depending on the skeletal site measured, which has a relatively slow rate of turnover [15]. In addition to BMD, several other parameters that can be measured during the same DXA scan may help to identify patients at high risk of fractures, such as the femoral neck length, the neck-diaphysis angle, the cross-sectional moment of inertia, and the cortical thicknesses. In addition, they reflect bone geometry or macroarchitecture, which is influenced by BMD [16]. To partially answer this problem the current osteoporosis classification criteria drafted by the World Health Organization (WHO) are currently revised to include clinical risk factors (http://www.shef.ac.uk/FRAX/).

## 4. Diabetes and Osteoporosis

Diabetes itself is associated with increased risk of fracture, although T2DM is often characterized by normal or high bone mineral density (BMD). Thus, diabetes may be associated with a reduction of bone strength, that is, not reflected in the measurement of BMD [17]. Diabetic osteopathy is a significant comorbidity of both forms of diabetes and is characterized by microarchitectural changes that decrease bone quality leading to an increased risk of bone fracture in both types of diabetes [18, 19].

T2DM is associated with an increased risk of hip fracture in both men, summary relative risk (RR) 2.8, and women, summary RR 2.1. Results are consistent between studies of men and women and between studies conducted in the United States and Europe. The association between type of diabetes and hip fracture incidence is stronger for T1DM, summary RR 6.3, than for T2DM, summary RR 1.7 [3]. In other meta-analyses, Vestergaard estimated a risk ratio for diabetes and hip fracture of 1.38 (95% CI, 1.25–1.53) for T2DM and 6.94 (95% CI, 3.25–14.78) for T1DM [17]. Increased risk of nonspine fractures in general has also been reported for T2DM [20].

The Nurses' Health Study with 109,983 women aged 34–59 years and followup of 22 years for the occurrence of hip fracture indicated that both type 1 and type 2 diabetes are associated with an increased risk of hip fracture. The results of this study highlight the need for fracture prevention strategies in all patients with diabetes [21].

### 4.1. The Pathogenesis of Bone Changes in Diabetes

Although the relationship between diabetes and osteoporosis has been widely investigated, it remains controversial. Diabetes could impact bone through several mechanisms, some of which may have contradictory effects.

The fracture risk of T1DM increases because of a decrease of BMD. In patients with T1DM the impaired bone formation is a result of absolute deficiency of insulin and insulin-like growth factor-1 (IGF-1), which leads to lower values of peak bone mass. In type 2 diabetes, obesity, increased load on bone, and insulin resistance resulting in hyperinsulinaemia lead to increased bone formation [22]. The coexistence of other autoimmune diseases with T1DM can lead to an additional risk factor for osteoporosis and increased fracture risk (secondary osteoporosis) in T1DM [23].

In both types of diabetes, bone displays inferior quality and strength [23]. T2DM reduces bone quality rather than BMD. Several risk factors for osteoporotic fractures are known and they are listed in Table 1.

#### 4.1.1. Hyperglycemia

Hyperglycemia resulting from impaired secretion and/or action of insulin acts on bone tissue cells through an increased production of interleukin-6 (IL-6) in osteoblast line cells. IL-6 stimulates osteoclasts to resorb bone. The accumulation of advanced glycation end products (AGEs) in collagen leads to inferior bone quality and strength. Furthermore, glycated collagen inhibits expression in osteoblasts [24]. The relationship between hyperglycemia and fracture risk does not appear to be linear. Studies have reported no increase in risk [25], or even decreased risk [26], comparing those with impaired glucose tolerance to those with normoglycemia. Another indirect effect of hyperglycemia is glycosuria, which causes hypercalciuria, leading to decreased levels of calcium in the body and poor bone quality, and fastens bone loss [27]. Among those with diabetes, there is not an established relationship between glycated hemoglobin (A1C) and fracture risk. Most observational studies have found no effect [28].

#### 4.1.2. Hypoglycemia

The risk of fractures in diabetes is also affected by the incidence of hypoglycemic episodes, if they especially are not preceded by prodromal symptoms. Although hypoglycemia can occur with sulfonylurea use, an increased risk of falls with low A1C levels is associated mainly with insulin use [29].

Hypoglycemic treatments could modulate the risk of fractures in many ways. Insulin-sensitizing treatment with metformin is not associated with a higher incidence of bone fractures [30]. Fracture rates are higher among all patients taking glitazones (TZDs). TZDs act as stimulators of the nuclear receptor peroxisome proliferator-activated receptor gamma (PPAR-*γ*) and could reduce bone density through the inhibition of osteoblast differentiation and activity. In fact, PPAR-*γ* activation induces the differentiation of multipotent mesenchymal stem cells into adipocytes, rather than osteoblasts, and increases osteoblast apoptosis [31, 32]. On the other hand, the insulin-sensitizing effect of TZDs reduces circulating insulin levels and therefore the insulin anabolic effect on the bone [18]. Higher incidence of fractures has been reported in insulin-treated patients in comparison with noninsulin-treated individuals [33]. In addition, the recently introduced class of incretin-based drugs (i.e., GLP-1 receptor agonists and DPP-4 inhibitors) is expected to exert potentially beneficial effects on bone health, possibly due to a bone anabolic activity of GLP-1 that can be either direct or indirect through the involvement of thyroid C cells [34]. C cells are mainly known for producing calcitonin, a hypocalcemic and hypophosphatemic hormone. Calcitonin suppresses resorption of bone by inhibiting the activity of osteoclasts. Some studies found that GLP-1 and other incretin hormones, such as GIP or GLP-2, could have positive effects on bone through antiresorptive and anabolic properties, suggesting beneficial effects of antidiabetic drugs like GLP-1R agonists or DPP-4 inhibitors on bone metabolism. The molecular mechanisms involved Wnt/beta-catenin pathway, OPG/RANKL ratio, and sclerostin levels [35].

#### 4.1.3. Insulin

Insulin is an anabolic hormone, which acts on bone through insulin receptors expressed by osteoblasts—IRS-1 and IRS-2 (insulin-like substrate). Stimulation of IRS-1 affects bone turnover, while stimulation of IRS-2 shifts the balance between bone formation and resorption towards the former. Insulin stimulates osteoblast proliferation, inactivates p27 (responsible for osteoblastogenesis), promotes collagen synthesis, and increases glucose uptake [18]. In T1DM, the deficiency of insulin and IGF-1, which is present since the diagnosis, leads to impaired bone formation, abnormal mineralisation, abnormal bone microarchitecture, increased fragility of the bone, and reduced peak bone mass [36]. In T2DM hyperinsulinism (the stimulatory effects of insulin on bone formation) coupled with insulin resistance increases bone mass through effects on bone formation via IRS-1 and IRS-2 surface receptors on osteoblasts and by reducing the concentration of sex-hormone binding globulin (SHBG), which leads to increased concentrations of estradiol and testosterone [37].

#### 4.1.4. Genetic Factors

Bone mineral density is affected by genetic factors. The A1-type-1-collagen (COL1A1) gene polymorphism in patients with T1DM is associated with reduced BMD at femoral neck and reduced serum vitamin D levels versus controls [38]. Vitamin D receptor gene polymorphism has also effect on BMD in diabetics [39].

#### 4.1.5. Alterations in Collagen Cross-Link Formation

Bone matrix consists of a two-phase composite material—the mineral phase (provides stiffness) and collagen fibers (provide tensile strength, ductility, and toughness) [40]. Collagen cross-linking plays an important role in bone strength [41]. Collagen cross-links can be divided into lysyl-hydroxylase- and lysyl-oxidase-mediated enzymatic immature divalent cross-links, mature trivalent cross-links, and glycation- or oxidation-induced nonenzymatic cross-links (AGEs) such as pentosidine [42]. These types of cross-links differ in the mechanism of formation and in function [43]. Not only hyperglycemia but also oxidative stress induces the reduction in enzymatic beneficial cross-links and the accumulation of disadvantageous AGEs in bone.

#### 4.1.6. Changes in Bone Turnover Markers (BTM)

Bone turnover is a dual relationship between the process of bone formation by osteoblasts (creation of new bone) and the process of bone resorption by osteoclasts (removal of old bone) [44]. Bone markers are subdivided into bone formation and bone resorption markers. Bone formation markers consist of osteocalcin (OC), bone-specific alkaline phosphatase (BAP), alkaline phosphatase (AP), procollagen type 1 amino terminal propeptide (P1NP), and procollagen type 1 carboxyl terminal propeptide (P1CP), while resorptive markers consist of N-terminal cross-linked telopeptide of type-I collagen (NTX) and C-terminal cross-linked telopeptide of type-I collagen (CTX) [45]. Several markers, especially OC, CTX, and P1NP, may also vary with blood glucose or glucose intake, making them perhaps less markers of bone turnover in diabetics and more markers of alterations in glucose metabolism. In most studies of bone turnover markers, osteocalcin, a marker of formation, is decreased with T2DM [46]. However, other formation markers are not consistently different in diabetic patients [47]. Resorption markers have been reported as increased, decreased, or not different in those with diabetes [48]. Another issue is kidney function, which may influence the measurement of several biochemical markers of bone turnover and also influence histomorphometry of the bone. The lack of a difference in bone turnover markers indicates that T1DM and T2DM are not different regarding the effect on bone markers, although Scl levels are higher in T2DM, proposing that bones are affected through an antagonizing effect on the WNT pathway in T2DM, but not in T1DM [49].

Osteocalcin (OC), one of the osteoblast-specific secreted proteins, has several hormonal features and is secreted in the general circulation from osteoblastic cells [50]. Recent animal studies have shown that uncarboxylated OC (ucOC) action is related to bone metabolism and glucose metabolism and fat mass [51, 52]. Pittas et al. have shown that serum OC concentration is inversely associated with fasting plasma glucose, fasting insulin, homeostasis model assessment for insulin resistance, high-sensitivity C-reactive protein, interleukin-6, body mass index, and body fat in cross-sectional analyses [53].

#### 4.1.7. Vitamin D

Most studies across a variety of geographic locations suggest that vitamin D insufficiency is more common in individuals with diabetes compared to the general population [54, 55]. Proposed mechanisms for vitamin D deficiency in diabetes include genetic predisposition (T1DM), increased BMI (T2DM), concurrent albuminuria (T1DM or T2DM), or exaggerated renal excretion of vitamin D metabolites or vitamin D-binding protein (T1DM, T2DM) [56, 57].

#### 4.1.8. Osteoprotegerin and RANK

Serum osteoprotegerin (OPG) is significantly increased in diabetic patients, prompting expanded investigation of the correlation between OPG production/release and glycemic levels [58]. Osteoprotegerin is a protein belonging to the family of tumour necrosis factor receptors (TNFR) capable of binding with receptor activator of nuclear factor kappa B ligand (RANKL), which prevents RANKL from binding to receptor activator of nuclear factor kappa B (RANK) and results in the suppression of osteoclastogenesis. Elevated osteoprotegerin in patients with T1DM may be the body's response to increased bone resorption [59]. Serum levels of OPG, but not of its cognate ligand receptor activator of nuclear factor kappa B ligand (RANKL), are significantly increased also in T2DM patients compared with healthy blood donors [58].

#### 4.1.9. Wnt Signaling Pathway

Wnt signaling is also thought to be a pathogenetic feature of osteoporosis in DM. In particular, Wnt signaling has been shown as an important regulatory pathway in the osteogenic differentiation of mesenchymal stem cells not only in the embryonic development but also in the maintenance and differentiation of the stem cells in adulthood. Induction of the Wnt signaling pathway promotes bone formation while inactivation of the pathway leads to osteopenic states. Activating and inactivating aberrations of the canonical Wnt signaling pathway in osteogenesis result in sclerosteosis and osteoporosis, respectively. Mani et al. have shown that a single missense mutation in low-density lipoprotein receptor-related protein 6, the coreceptor for the Wnt signaling pathway, is genetically linked to osteoporosis as well as DM, dyslipoproteinemia, and coronary artery disease [60]. In addition, several studies have documented that T-cell-specific transcription factor- (TCF-) 4, the partner of *β*-catenin in the canonical Wnt signaling pathway, is the strongest T2DM susceptibility gene [61–63].

#### 4.1.10. Obesity and BMI

A low BMI is associated with the decreased BMD, the increased possibility of osteoporosis, and the risk of fracture [64]. A meta-analysis demonstrated that BMI is also an important predictor of BMD in T2DM [19]. Overweight and obesity are believed to be protective factors of BMD [65, 66]. Obesity, widespread in T2DM, is strongly associated with higher BMD probably through mechanical loading and hormonal factors including insulin, estrogen, and leptin [67]. Recently published results from the Global Longitudinal Study of Osteoporosis in Women (GLOW) demonstrated that association between fracture risk, height, weight, and BMI differs according to fracture site—there is an inverse linear association between BMI and wrist fractures, positive linear association between BMI and spine fractures, and no significant association between BMI and upper leg fractures [68].

#### 4.1.11. Complications of DM

A few studies have reported on diabetes-related complications as risk factors of fracture in those with T2DM, but results have not been consistent. The development of osteoporosis in both types of diabetes is also promoted by the coexistence of chronic microvascular complications, which also affect the bone marrow blood vessels [69].

### 4.2. Problems in Clinical Practice

Mainly older adults with T2DM are more likely to fall, but little is known about risk factors of falls in this population [70]. A higher risk of falls and the resulting fractures in patients with diabetes may result from the presence of diabetic retinopathy or cataracts, which impair visual acuity. In patients with coexisting sensory motor neuropathy and diabetic foot, balance disorders and falls are also observed [56]. Patel et al. suggest that reduced vibration perception (a measure of peripheral neuropathy) is an important risk factor for falling. The authors conclude also that quantitative ultrasound (QUS), as opposed to DXA, may be a more useful method for fracture risk prediction in older women with type 2 DM [71]. Insulin therapy is also associated with increased falls, possibly because of more severe disease and/or hypoglycemic episodes [72].

In T1DM, the increased risk of fractures may result from reduced BMD, so the basic diagnostic procedure and gold standard for diagnosis of osteoporosis and risk groups of diabetic patients are DXA. In T2DM the higher risk for osteoporotic fractures may be a consequence of poorer bone quality, impaired micro- and macroarchitecture, and increased tendency to fall. There is a need to clarify the use of standard methods for assessing fracture risk in T2DM.

The 10-year absolute risks of hip and osteoporotic fracture can be calculated using the FRAX algorithm. The FRAX scores are designed to predict the absolute 10-year risks of hip and osteoporotic fracture using hip BMD and other clinical risk factors of fracture [73]. The FRAX algorithm includes femoral neck BMD *T*-score, age, sex, body mass index, previous history of fracture, parental history of hip fracture, current smoking, recent use of corticosteroids, presence of rheumatoid arthritis, and at least 3 alcoholic beverages per day [73–75]. Schwartz et al. showed in a study that femoral neck BMD *T*-score and FRAX score are both associated with fracture risk in older adults with T2DM and both methods appear to be useful for clinical evaluation of fracture risk. They also warn that, at any given *T*-score or FRAX score, fracture risk was higher in those with diabetes [76].

To improve the management of osteoporosis, bone turnover biomarkers can be used. They can assess, directly or indirectly, bone development or bone resorption activity. According to the level of bone turnover we can estimate the fracture risk and evaluate the effect of treatment. These markers are measured in serum, plasma, and urine [77]. The ability to measure these markers has led to major advances in clinical research. Unfortunately, for reasons of availability, cost, and reproducibility, biological markers of bone turnover are not commonly measured among nonspecialists of bone diseases.

#### 4.2.1. Bone Quality

Bone must be stiff and able to resist deformation, so that loading is possible. Bone must also be flexible and able to deform to allow energy absorption during impact loading. Bone must also be light to allow movement [78]. The balance between bone's material stiffness and its flexibility is achieved by varying its mineral content. The greater the mineral content, the greater the material stiffness and the lower the flexibility [26]. Bone strength, one of its major determinants, is dependent both on bone mass, reflected by bone mineral density (BMD), and on bone microarchitecture [79]. Thus, bone strength arises from both bone quantity and bone quality. Bone quality encompasses the geometric and material factors that contribute to fracture resistance [80]. Bone quality is not precisely defined. It is described as an amalgamation of all the factors that determine how well the skeleton can resist to fractures, such as the microarchitecture of the bone, the accumulated microscopic damages, the quality of collagen, the size of mineral crystals, and the rate of bone turnover [16, 26].

In fact, BMD explains only 70–75% of the variance in bone strength, while the rest could be related to other factors such as the accumulation of microfractures, the altered bone microarchitecture, the disordered bone remodeling, or the influence of extraskeletal risk factors [81].

Diabetes showing hyperglycemia and oxidative stress deteriorates bone material properties in terms of collagen posttranslational modification such as enzymatic immature and mature cross-links and nonenzymatic AGEs formation. The adverse effects of AGEs on bone cells accelerate bone fragility and impaired bone quality in diabetes [27].

Despite the use of BMD, biomarkers, and fracture clinical risk factors, many patients at risk for fractures are not detected and many fractures are not explained. BMD is only an assessment of bone mass. It does not provide information on bone quality, another key parameter describing bone. Fracture clinical risk factors (FRAX tool) are an indirect assessment of bone quality [82].

The presence of prevalent vertebral fractures (VFs) could also be used for the assessment of bone quality in individual patients, because a large study on the incidence of VFs in postmenopausal osteoporosis has shown that patients with previous VFs were more likely to suffer from new VFs [83, 84].

One important way to describe bone quality is to assess its microarchitecture. Bone microarchitecture contributes to the mechanical strength of bone and, thus, to its ability to withstand fractures. Bone loss is often accompanied by deterioration in bone architecture, resulting from a decrease in the number of trabeculae of cancellous bone, increased intertrabecular distances, and a loss of trabecular connectivity. In addition, a reduction in the thickness of cortical bone and an increase in its porosity of trabecular bone can result in fragility of the femoral neck [85]. Osteoporotic bone is, hence, called “porous.” Although no single method can completely characterize bone quality, current noninvasive imaging techniques can be combined with ex vivo mechanical and compositional techniques to provide a comprehensive understanding of bone quality [86].

A variety of imaging techniques allow characterization of bone geometry and microarchitecture from the macroscale to the microscale and also to nanoscale. Methods for characterizing bone geometry and microarchitecture include quantitative CT, high-resolution peripheral quantitative CT, high-resolution MRI, and micro-CT. Macroscopic assessment of three-dimensional (3D) bone geometry can be performed in vivo using quantitative CT (QCT), but an important drawback of QCT is its delivery of ionizing radiation to patients [87]. The advent of high-resolution peripheral QCT (HR-pQCT) scanners with isotropic resolution of approximately 80 *μ*m has enabled in vivo imaging of 3D trabecular morphology at peripheral sites such as the distal radius. These measurements are largely restricted to peripheral sites but have the concomitant benefit of reduced radiation doses relative to those from whole-body QCT scans [88, 89]. High-resolution MRI (HR-MRI) allows nonionizing 3D imaging of the trabecular network at peripheral sites. A critical advantage of this technique is its ability to generate 3D images of bone geometry and microarchitecture without ionizing radiation, but the disadvantages include the long scan times required for high-resolution images of trabecular bone [90].

To meet the need for a clinical tool capable of assessing bone microarchitecture, the trabecular bone score (TBS) was developed [91, 92].

#### 4.2.2. Trabecular Bone Score

Trabecular bone score (TBS) is a novel noninvasive modality designed to assess the trabecular microarchitecture parameters derived from DXA images. The proponents described the TBS as a texture parameter that reflects pixel gray level variations in DXA images [93]. These variations may reflect microarchitecture, but the pixel size of currently available DXA machines is about four times larger than the mean trabecular size. TBS development is based on the following facts [94–96].

- A healthy patient has well- and dense-structured trabecular bone at the vertebral level (high connectivity, high trabecular number, and small spaces between trabeculae). If we project this structure onto a plane, we obtain an image containing a large number of pixel value variations, but the amplitudes of these variations are small.
- Conversely, an osteoporotic patient has an altered and porous trabecular bone structure (low connectivity, low trabecular number, and wide spaces between trabeculae). If we project this structure onto a plane, we obtain an image containing a low number of pixel value variations, but the amplitudes of these variations are high.

The amount of trabecular bone lost during aging in women and men is similar or only slightly less in men than women [97]. Strength of the vertebrae is compromised more by loss of connectivity than by trabecular thinning [98].

TBS measures the level of degradation of trabecular structure. We get a result, which can be evaluated according to Table 2 (temporary consensus available on the website: http://www.medimapsgroup.com/).

Large retrospective study with more than 29,000 postmenopausal women showed that TBS independently predicts fractures in a subpopulation of patients with diabetes [99].

We performed a retrospective cohort study using BMD results from clinical registry of our department. We evaluated the ability of lumbar spine TBS to account the increased risk of fractures in T2DM in 56 postmenopausal women patients with T2DM and 61 women patients without DM or IGT. T2DM was associated with higher BMD (1.155 versus 1.048 g/cm^2^ in average, *P* < 0.05) at all sites but lower lumbar spine TBS (1.211 versus 1.295 in average, *P* < 0.05). The adjusted odds ratio (aOR) for a measurement in the lowest versus the highest tertile was less than 1 for BMD (*P* < 0.05) but was increased for lumbar spine TBS (aOR 2.39, 95% confidence interval (CI) 2.22–2.81). Also according to these results lumbar spine TBS predicts osteoporotic fractures in those with diabetes and captures a larger portion of the diabetes-associated fracture risk than BMD [100].

From a clinical point of view TBS is able to predict future fracture risk [101], in combination with BMD. Using TBS we can increase the number of patients with a well identified risk, to improve the management of patients in which bone quality has a greater impact than bone quantity [102–104]. This method allows us to follow the evolution of a patients' trabecular bone texture over time and to monitor the effects of antiresorptive or anabolic treatment [105, 106].

TBS can be used in diabetology as a diagnostic toll and it can be used also to evaluate the effect of treatment. It is necessary to keep in mind that TBS is not intended to replace existing tools but rather to supplement them and assist clinicians in our medical decisions. BMD and TBS are two independent parameters reflecting different bone properties: quantity and quality, respectively. Both BMD and TBS are very important in assessing bone strength. Bone strength status is important to evaluate the risk of fracture as well as to make decision about the type of treatment [107].

## 5. Conclusions

Given the current data, which suggest that diabetic patients are at a higher risk of fracture, it would be reasonable to screen diabetic patients for osteoporosis. The current osteoporosis guidelines for screening can be used for patients with T1DM and also T2DM, but it is important to bear in mind that DM is a risk factor for osteoporosis and fracture and that fracturing can occur at higher BMD levels in patients with DM [108].

As a result of the ineffectiveness of BMD in assessing fracture risks in T2DM, the major clinical problems are how to assess the risks and when to start therapy for preventing fractures in daily practice. Although there are potential candidates (osteocalcin, AGEs, and insulin) for such purposes, it is unclear whether or not they could predict the occurrence of new fractures in T2DM patients in a prospective fashion and can be used in daily practice.

A simple and recommended procedure for all physicians who are engaged in T2DM treatment is to question the patients about their fracture histories. It is likely that about half of them will be identified as those who have bone fragility and need osteoporosis treatment for fracture prevention. Also if T2DM patients undergo spinal X-ray examination we should directly look for vertebral fractures.

Recently, the fracture risk assessment (FRAX) algorithm has been developed by the WHO, which could assess the fracture risk of an individual even if BMD is not measured [109]. This algorithm integrates the influence of several well-validated risk factors for fractures that are independent of BMD and therefore it might be useful for the case-finding strategy that identifies diabetic patients at high risk for fracture.

The TBS is a new parameter, that is, determined from grey level analysis of DXA images. The TBS meets the need for a noninvasive method for assessing bone microarchitecture—key determinant of bone strength. In addition, the TBS can be estimated very simply, using widely available DXA machines and during the same procedure as BMD measurement. The TBS is a quantitative value, that is, reproducible and easy to handle.

Therapeutic considerations in diabetic patients with osteoporosis are recommendations from good clinical practice, rather than evidence-based studies (Table 3).

All patients with DM should be counseled regarding their risk of osteoporosis and fractures. They should also be advised on adequate calcium (at least 100–1200 mg/day) and vitamin D (800–1000 IU/day) intake [110, 111]. When a patient meets guidelines for treatment, there are several options including antiresorptive medications such as bisphosphonates, denosumab, SERM, and anabolic agent teriparatide. There are no long-term data on the effectiveness of either of these types of medications in patients with diabetes [112]. Achieving adequate glycaemic control in patients with diabetes is especially important as there are data to suggest that the microvascular complications of DM, such as retinopathy and neuropathy, which arise from less than ideal glycaemic control, can lead to falls and subsequent fractures.

## Figures and Tables

**Table 1:** Risk factors for osteoporotic fractures in diabetes (modified according to [23]).

Risk for osteoporosis	

Directly due to diabetes	
(i) Diabetes mellitus types 1 and 2	
(ii) Poor glycemic control and hyperglycemia	
(iii) Hypoglycemia (due to DM treatment)	
Due to complications of diabetes	
(i) Nephropathy and other kidney diseases	
(ii) Neuropathy	
(iii) Diabetic diarrhea	
Due to diseases associated with diabetes	
(i) Thyroid gland dysfunction (Grave's disease)	
(ii) Intestinal bowel diseases and celiac sprue	
(iii) Amenorrhea	
(iv) Delayed puberty	
(v) Eating disorders	

Risk of falls	
(i) Episodes of hypoglycemia due to medication (mostly insulin)	
(ii) Episodes of nocturia (during uncompensated DM)	
(iii) Poor vision due to retinopathy or cataracts	
(iv) Poor balance due to neuropathy, foot ulcers, or amputations of diabetic foot	
(v) Orthostatic hypotension (due to cardiac autonomic neuropathy)	
(vi) Impaired joint motility due to cheiropathy and arthropathy	

**Table 2:** Levels of degradation of trabecular structure according to TBS.

Degradation	Description	TBS range
Normal		Above 1.350

Moderate	Grade 1	1.300–1.350
	Grade 2	1.250–1.300
	Grade 3	1.200–1.250

Degraded	Severe	1.100–1.200
	Highly degraded	Below 1.100

**Table 3:** General management of osteoporosis in diabetic patients.

(i) To avoid glitazones	
(ii) Good glycaemic control	
(iii) Minimizing of hypoglycemic episodes	
(iv) Prevention of diabetic complications, especially kidney disease	
(v) To assess and prevent falls	
(vi) Supplementation with calcium and vitamin D	
(vii) Specific antiporotic medication (antiresorptive or osteoanabolic treatment)	
