# Corrigendum to “Osteoporosis, Fractures, and Diabetes”

**DOI:** 10.1155/2017/2846080

**Published:** 2017-05-30

**Authors:** Peter Jackuliak, Juraj Payer

**Affiliations:** 5th Department of Internal Medicine, Comenius University Faculty of Medicine and University Hospital in Bratislava, Ruzinovska 6, 826 06 Bratislava, Slovakia

In the article titled “Osteoporosis, Fractures, and Diabetes” [1], there was an error regarding the FRAX® tool, which should be clarified as follows:

The article notes: “To partially answer this problem the current osteoporosis classification criteria drafted by the World Health Organization (WHO) are currently revised to include clinical risk factors (http://www.shef.ac.uk/FRAX/)” and “Recently, the fracture risk assessment (FRAX) algorithm has been developed by the WHO, which could assess the fracture risk of an individual even if BMD is not measured [109].” However, the World Health Organization (WHO) did not develop, test, or endorse the FRAX^®^ tool or its recommendations [2]. The metabolic bone disease unit at the University of Sheffield that developed FRAX was a WHO Collaborating Centre from 1991 to 2010, but treatment guidelines must undergo a formal process before they can be endorsed by the WHO.

## References

[1] Jackuliak P., Payer J. (2014). Osteoporosis, fractures, and diabetes. *International Journal of Endocrinology*.

[2] Ford N., Norris S. L., Hill S. R. (2016). Clarifying WHO’s position on the FRAX® tool for fracture prediction. *Bulletin of the World Health Organization*.

